# Histone H4 expression is cooperatively maintained by IKKβ and Akt1 which attenuates cisplatin-induced apoptosis through the DNA-PK/RIP1/IAPs signaling cascade

**DOI:** 10.1038/srep41715

**Published:** 2017-01-31

**Authors:** Ruixue Wang, Xuelian Zheng, Lei Zhang, Bin Zhou, Huaizhong Hu, Zhiping Li, Lin Zhang, Yong Lin, Xia Wang

**Affiliations:** 1 Laboratory of Molecular and Translational Medicine, Key Laboratory of Birth Defects and Related Diseases of Women and Children (Sichuan University) of Ministry of Education, Department of Obstetrics and Gynecology, West China Second University Hospital, Sichuan University, Chengdu 610041, China; 2 Department of Abdominal Oncology, Cancer Center, West China Hospital, Sichuan University, Chengdu 610041, China; 3 Department of Immunology, West China School of Preclinical and Forensic Medicine, Sichuan University, Chengdu 610041, China; 4 Molecular Biology and Lung Cancer Program, Lovelace Respiratory Research Institute, 2425 Ridgecrest Dr., SE., Albuquerque NM 87108, USA

## Abstract

While chromatin remodeling mediated by post-translational modification of histone is extensively studied in carcinogenesis and cancer cell’s response to chemotherapy and radiotherapy, little is known about the role of histone expression in chemoresistance. Here we report a novel chemoresistance mechanism involving histone H4 expression. Extended from our previous studies showing that concurrent blockage of the NF-κB and Akt signaling pathways sensitizes lung cancer cells to cisplatin-induced apoptosis, we for the first time found that knockdown of Akt1 and the NF-κB-activating kinase IKKβ cooperatively downregulated histone H4 expression, which increased cisplatin-induced apoptosis in lung cancer cells. The enhanced cisplatin cytotoxicity in histone H4 knockdown cells was associated with proteasomal degradation of RIP1, accumulation of cellular ROS and degradation of IAPs (cIAP1 and XIAP). The cisplatin-induced DNA-PK activation was suppressed in histone H4 knockdown cells, and inhibiting DNA-PK reduced expression of RIP1 and IAPs in cisplatin-treated cells. These results establish a novel mechanism by which NF-κB and Akt contribute to chemoresistance involving a signaling pathway consisting of histone H4, DNA-PK, RIP1 and IAPs that attenuates ROS-mediated apoptosis, and targeting this pathway may improve the anticancer efficacy of platinum-based chemotherapy.

DNA-damaging chemotherapy agents such as cisplatin are components of the first-line treatment regimes for many solid tumors. Cisplatin kills cancer cells mainly through induction of DNA damage-mediated apoptosis. By binding to guanine and adenine in DNA, cisplatin induces various types of DNA damages including interstrand and intrastrand crosslink, DNA-protein crosslink, and replication-associated DNA double strand break^1^,^2^. While moderate DNA damage can be repaired through mechanisms including nucleotide excision repair (NER) and mismatch repair (MMR), extensive DNA damage elicits signals to activate apoptosis to eliminate damaged cells. Thus, potentiation of cisplatin-induced apoptosis pathway may be exploited for sensitization of platinum-based chemotherapy.

Although initial responsiveness to platinum-based therapy is commonly achieved, most patients develop drug resistance and disease eventually relapses. The resistance to cisplatin in tumor cells is believed to be multifactorial, which includes changes in cellular uptake and efflux of the drug, increased detoxification of the drug, impaired DNA repair, dysfunction of the apoptotic-signaling pathways, and activation of cell survival pathways^1^,^2^. NF-κB and Akt are two major survival pathways in cells. Accumulating reports have shown that both constitutive and chemotherapeutic-induced activations of NF-κB and Akt are involved in chemoresistance. NF-κB activates expression of certain genes that are responsible for cell proliferation and survival^3^. Akt is associated with oncogenic cell transformation and the maintenance of malignant phenotypes of cancer cells. Consistently, targeting NF-κB or Akt has been shown to enhance the anticancer activity of chemotherapeutics in a variety of cancer cells^4^,^5^,^6^,^7^. However, the defined mechanism, particularly the cooperation of these two pathways in chemoresistance has not been well elucidated.

We found in our recent studies that concurrent blockage of the NF-κB and Akt signaling pathways effectively sensitizes lung cancer cells to apoptosis induced by chemotherapeutics including cisplatin in both cell culture and mouse xenograft tumor models^8^,^9^,^10^,^11^. To explore the mechanism underlying the cooperation of the NF-κB and Akt pathways in chemoresistance, a gene expression microarray assay was performed in cells with stable knockdown of Akt1, the NF-κB-activating kinase IKKβ or both of them. The expression of all the histone H4-coding genes was down-regulated when Akt1 and IKKβ were concurrently knocked down. These findings suggest that histone H4 (referred as H4 hereafter), one of the core components of nucleosomes essential for chromatin structure, chromosome replication and gene transcription, could be a novel target of NF-κB and Akt for chemoresistance.

Histones are highly conserved and positively charged alkaline proteins that package the lengthy genomic DNA into the relatively small nucleus^12^. Approximately 146 bp of DNA is wrapped by an octamer consisting of two molecules of each of the four core histones (H2A, H2B, H3, and H4) to form nucleosomes, and the linker histone H1 interacts with linker DNA between nucleosomes to compact chromatin into higher order structures^12^,^13^. Expression of histone genes is regulated at transcriptional and post-transcriptional levels along the cell cycle^14^. Emerging studies have focused on histone post-translational modifications (PTMs) including acetylation, methylation, phosphorylation, ubiquitination, citrullination, sumoylation, biotinylation and ADP ribosylation in regulation of chromatin structure and gene expression^13^. Histone PTMs were also found to play an important role in carcinogenesis and cancer cell’s response to chemotherapy and radiotherapy^15^,^16^,^17^,^18^. However, little is known about the effect of histone expression level on chemoresistance^17^,^19^. Thus, we investigated whether H4 expression is involved in cisplatin chemoresistance in lung cancer cells. The results establish a novel mechanism by which NF-κB and Akt contribute to chemoresistance: to maintain histone H4 expression for activating the DNA-PK/RIP1/IAPs signaling cascade that attenuates ROS-mediated apoptosis. Targeting this signaling cascade may improve the anticancer efficacy of platinum-based chemotherapy.

## Results

### Concurrent knockdown of Akt1 and IKKβ down-regulates the expression of histone H4

**Figure 1:**
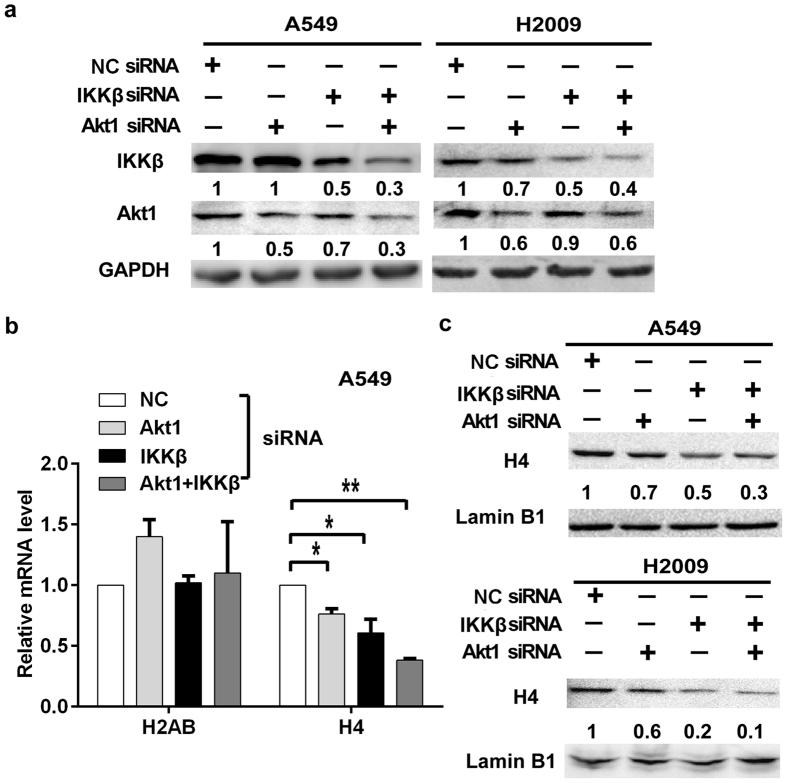
Concurrent knockdown of Akt1 and IKKβ down-regulates histone H4 expression. (**a**) A549 and H2009 cells were transfected with Akt1-, IKKβ-siRNA or both. Negative control (NC) siRNA transfected cells were used as a negative control. The knockdown efficacy of IKKβ and Akt1 was confirmed by Western blotting. GAPDH was used as a loading control. (**b**) Reduced histone H4 mRNA expression was detected by qRT-PCR. H2AB was detected as a negative control (*p < 0.05; **p < 0.01). (**c**) The expression of H4 protein was detected by Western blot. Lamin B1 was detected as a loading control.

Our previous studies have found that concurrently blocking the NF-κB and Akt pathways synergistically sensitized A549 cells to cisplatin-induced cell death *in vitro* as well as *in vivo*^8^,^9^. To explore the mechanism of which, cDNA microarray-base screening expression-altered genes was conducted. Among the genes changed the most, all the histone H4-coding genes were found to be down-regulated in expression when either Akt1 or IKKβ was suppressed. Concurrently knockdown of Akt1 and IKKβ cooperatively reduced H4 expression (Supplementary Table 1). This observation was validated by quantitative real-time RT-PCR and Western blot in two lung cancer cell lines, A549 and H2009, having siRNA-mediated knockdown of Akt1 and IKKβ expression individually or concurrently. The reduction of Akt1 or IKKβ expression as well as NF-κB and Akt activity was confirmed in these cells (Fig. 1a and data not shown). As shown in Fig. 1b, downregulation of H4 mRNA was detected when the expression of either Akt1 or IKKβ was suppressed, which was more prominent when Akt1 and IKKβ were concurrently knocked down. In contrast, the transcription of histone H2AB was not inhibited, substantiating the specificity of regulation of H4 by Akt1 and IKKβ. The expression of H4 protein was significantly decreased after knockdown of Akt1 or IKKβ (Fig. 1c). Consistent with the qRT-PCR result, concurrent knocking down Akt1 and IKKβ exerted a more striking inhibitory effect on H4 protein expression in A549 and H2009 cells (Fig. 1c). These results suggest that the NF-κB and Akt pathways cooperatively regulate H4 expression.

### Down regulation of H4 expression potentiates cisplatin-induced apoptosis

**Figure 2:**
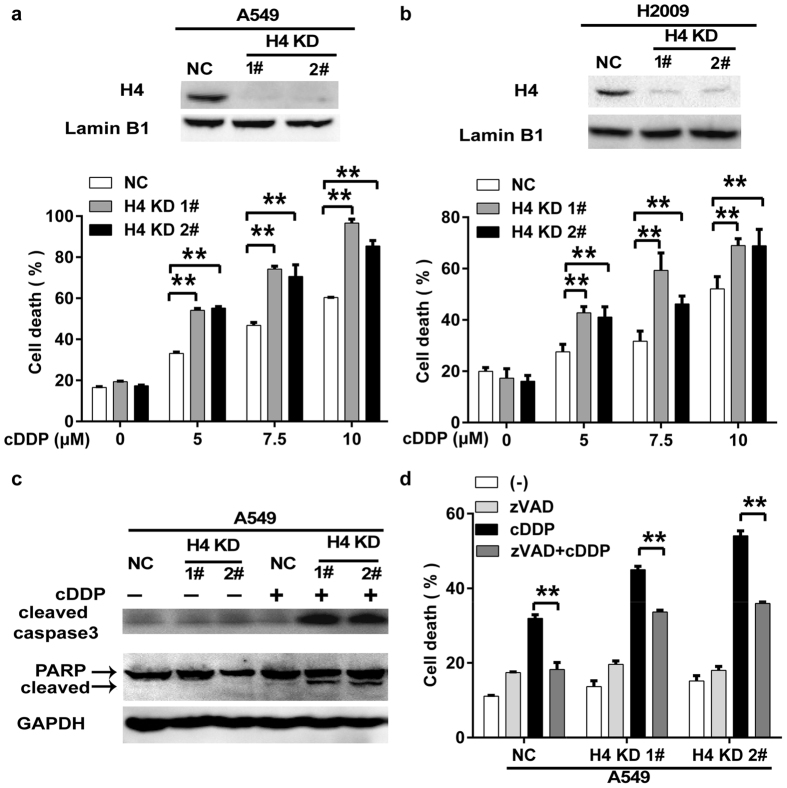
H4 knockdown sensitizes cancer cells to cisplatin-induced apoptosis. (**a,b**) The H4 knockdown cell clones (A549-H4 KD 1# and 2#, and H2009-H4 KD 1# and 2#) were established. H4 protein expression was detected by Western blot. The cells were treated with indicated concentrations of cisplatin. Cell death was measured by LDH releasing assay 72 h after cisplatin treatment. (**c**) Negative control (NC) A549 cells and its H4 knockdown clones were treated with cisplatin (7.5 μM) for 30 h. Active caspase-3 and PARP were detected by Western blot. GAPDH was detected as a loading control. (**d**) The cells were pretreated with zVAD-fmk (20 μM) for 1 h or remained untreated, and then treated with cisplatin (7.5 μM) for 72 h. Cell death was measured by LDH releasing assay. Columns, mean of three experiments; bars, SD. **p < 0.01.

To investigate if downregulation of H4 was involved in response to cisplatin, A549 and H2009 cell clones with stable H4 knockdown (A549-H4 KD and H2009-H4 KD) were established (Fig. 2a,b). H4 knockdown significantly increased cisplatin-induced cytotoxicity in both A549 and H2009 cells compared to that in respective control cells (Fig. 2a,b). Because H4 is the core histone of nucleosome, the proliferation of the cells was examined to exclude the impact of cell proliferation on cisplatin response. There was marginal effect of H4 inhibition on cell proliferation detected by CCK8 and flow cytometry assays (Supplementary Figs S1, S2), suggesting that the reduced H4 expression level was still sufficient for supporting cell proliferation and the effect of H4 KD on cisplatin-induced cell death was not directly associated with cell proliferation. To explore the mechanism of H4 suppression-mediated enhancement of cisplatin toxicity, apoptosis was examined with two distinct approaches. By Western blot, significantly enhanced activation of caspase 3 and cleavage of PARP in cisplatin-treated H4 KD cells were detected, comparing with that in cisplatin-treated control cells (Fig. 2c). With the pan-caspase inhibitor zVAD-fmk to block apoptosis, cisplatin-induced cytotoxicity was significantly suppressed in H4 KD cells (Fig. 2d). These results suggest that the enhanced sensitivity to cisplatin in H4 knockdown cells was associated with potentiated apoptosis activation.

### Increased cellular ROS accumulation contributes to potentiated cisplatin cytotoxicity in H4 KD cells

**Figure 3:**
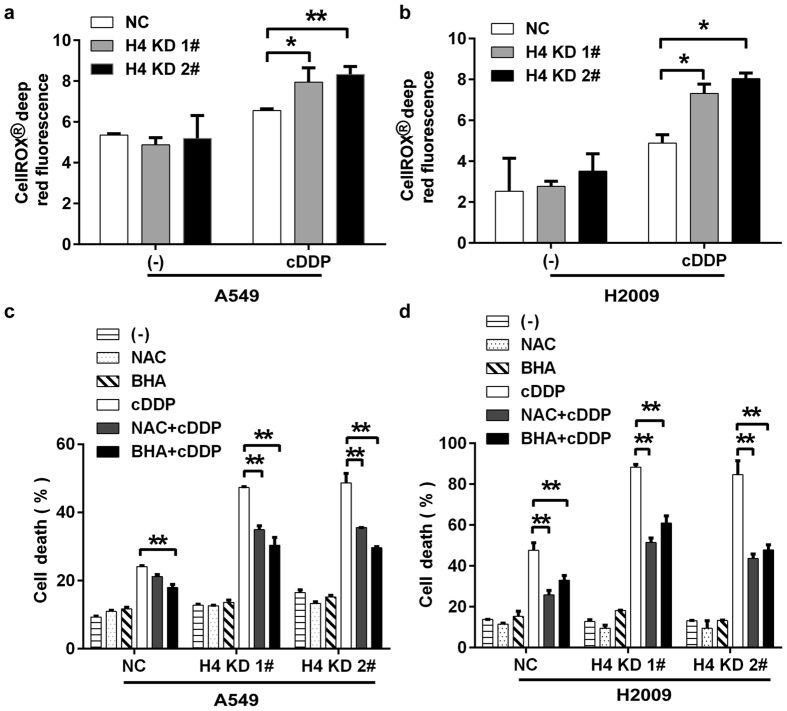
Cisplatin-induced cellular ROS accumulation contributes to the increased cytotoxicity in H4 KD cells. (**a,b**) The cells were treated with cisplatin (7.5 μM) for 7 h followed by incubating with CellRox dye for 30 min. Cellular fluorescence was measured with the Varioskan Flash Multimode Reader. (**c,d**) The cells were pre-treated with NAC (1 mM) or BHA (100 μM) for 1 h, followed by cisplatin (7.5 μM) treatment for another 72 h. Cell death was measured by LDH releasing assay. Columns, mean of three experiments; bars, SD. ^*^p < 0.05; **p < 0.01.

A growing body of evidence has shown that reactive oxygen species (ROS) contributes to activation of cell death pathways induced by chemotherapeutics^20^,^21^,^22^. Although the basal ROS level was not changed, cisplatin-induced cellular ROS was effectively increased in H4 stable knockdown cells (Fig. 3a,b). To address the role of ROS in enhanced cytotoxicity in cisplatin-treated H4 KD cells, the ROS scavengers butylated hydroxyanisole (BHA) and N-acetyl cysteine (NAC) were used to block ROS accumulation. Both BHA and NAC, which effectively inhibited cisplatin-induced accumulation of ROS (Supplementary Fig. S3a), significantly attenuated cisplatin-induced cell death (Fig. 3c,d). These results suggest that increased ROS accumulation contributed to enhanced cisplatin sensitivity in H4 KD cells.

### Histone H4 suppresses proteasomal degradation of RIP1 and IAPs induced by cisplatin

**Figure 4:**
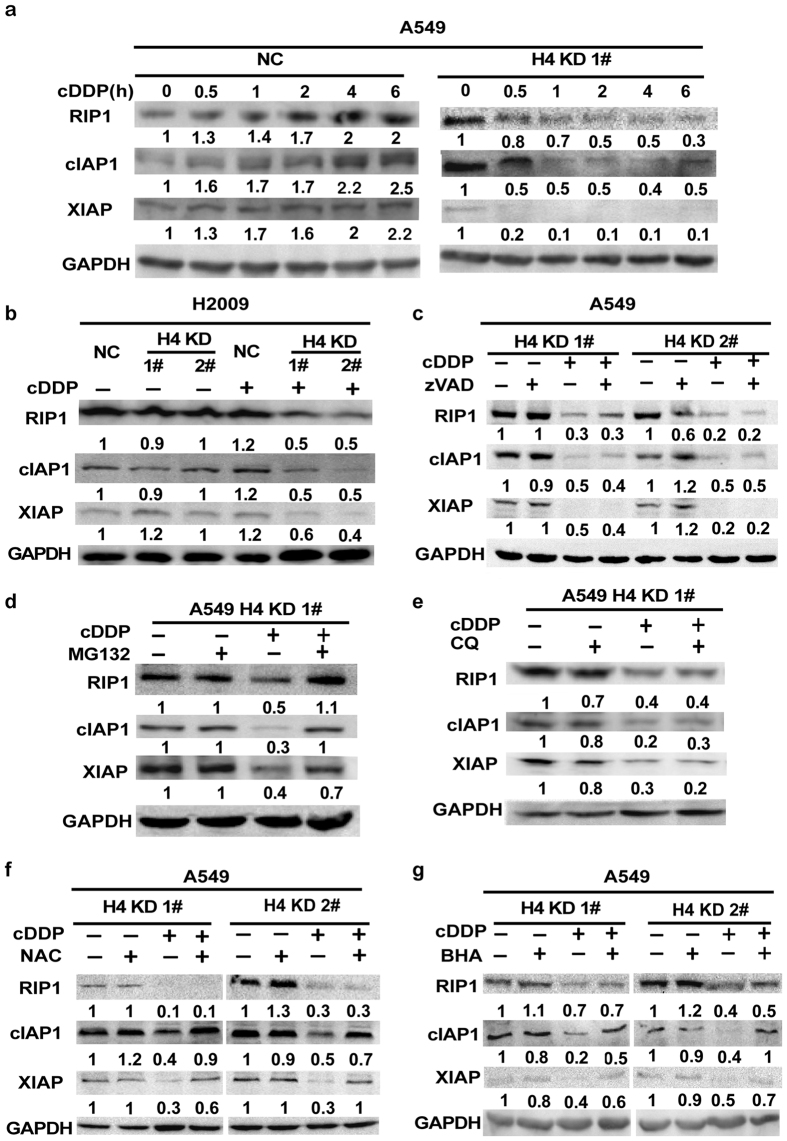
Histone H4 suppresses proteasomal degradation of RIP1 and IAPs induced by cisplatin. (**a**) The cells were treated with cisplatin (7.5 μM) for indicate times, and the expression of the indicated proteins was detected by Western blot. GAPDH was detected as a loading control. (**b**) The cells were treated with cisplatin (7.5 μM) for 2 h. The expression of the indicated proteins was detected by Western blot. GAPDH was detected as a loading control. (**c–g**) The cells were pretreated with zVAD (20 μM), MG132 (10 μM), chloroquine (CQ, 20 μM), NAC (1 mM) or BHA (100 μM) for 1 h. Then the cells were treated with cisplatin (7.5 μM) for another 2 h. The expression of the indicated proteins was detected by Western blot. GAPDH was detected as a loading control.

Our previous studies found that RIP1 suppresses cisplatin-induced ROS accumulation to retain expression of inhibitors of apoptosis family proteins (IAPs)^23^. Thus, we examined whether RIP1 and IAPs are involved in the cisplatin sensitivity increase in H4 KD cells. Cisplatin treatment caused a significant increase in the expression of RIP1, cIAP1 and XIAP in the control cells, which implies that these proteins may be involved in counteracting the apoptosis effect of cisplatin in wild-type cancer cells. However, an opposite trend of changes, a dramatic reduction of these proteins was seen when H4 was suppressed in both A549 and H2009 cells (Figs 4a,b, and S3b). zVAD was unable to restore RIP1 and IAPs expression in cisplatin-treated H4 KD cells (Fig. 4c), suggesting that the expression reduction of RIP1 and IAPs was not the result of apoptosis. Furthermore, the proteasome inhibitor MG-132, but not the lysosome inhibitor chloroquine, effectively restored RIP1 and IAPs expression in cisplatin-treated H4 KD cells (Figs 4d,e, S3c, and S3d), suggesting that H4 retains RIP1 and IAPs expression mainly through suppressing cisplatin-induced proteasomal degradation. To delineate the mechanism of ROS accumulation and expression of RIP1 and IAPs, the ROS scavengers BHA and NAC were used to pre-treat cells before cisplatin exposure in H4 KD cells. These ROS scavengers efficiently attenuated cisplatin-induced IAPs reduction, while did not impact the effect of cisplatin on RIP1 protein (Fig. 4f,g). Together with our previous report that RIP1 is upstream of ROS in cisplatin-treated cancer cells^23^, these results suggest that RIP1 reduction contributes to enhancement of cisplatin-induced ROS accumulation, which in turn, leads to degradation of IAPs in H4 KD cells.

### Degradation of RIP1 and IAPs contributes to increased cisplatin sensitivity

To further determine the role of RIP1 and IAPs in increased cisplatin sensitivity, over-expression of RIP1, cIAP1, or XIAP in H4 KD cells was conducted before cisplatin exposure. pR3RED was used as a marker for identification of transfected cells. Ectopic expression of RIP1, cIAP1 or XIAP effectively rescued H4 KD cells from the cytotoxic effects of cisplatin (supplementary Fig. S4), suggesting that down-regulation of RIP1 and IAPs is responsible for increased cisplatin sensitivity in H4 KD cells.

### Suppression of DNA-PK activation is responsible for RIP1 and IAPs downregulation in H4 knockdown cells

**Figure 5:**
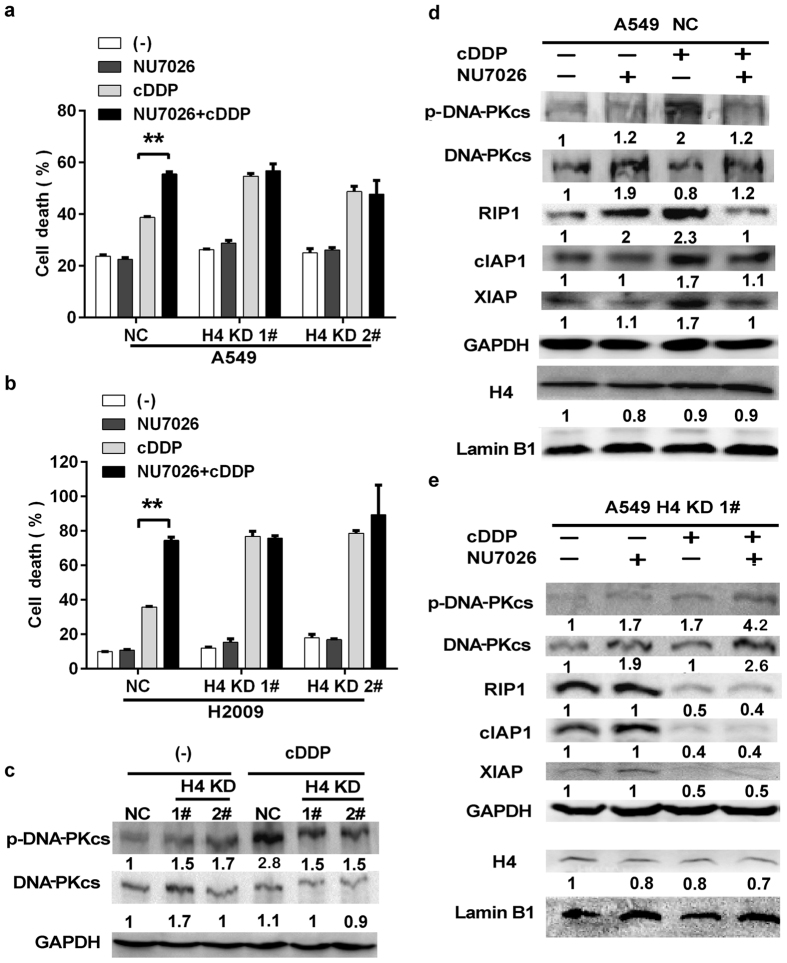
Suppression of cisplatin-induced DNA-PK activation contributes to RIP1 and IAPs downregulation in H4 knockdown cells. (**a,b**) The cells were pre-treated with DNA-PK inhibitor NU7026 (10 μM) for 1 h or left untreated and treated with cisplatin (7.5 μM) treatment for another 72 h. Cell death was measured by LDH releasing assay. Columns, mean of three experiments; bars, SD. **p < 0.01. (**c**) The cells were treated with cisplatin (7.5 μM) for 30 min. The expression of the indicated proteins was detected by Western blot. GAPDH was detected as a loading control. (**d,e**) The cells were pre-treated with NU7026 (10 μM) for 1 h or left untreated followed by cisplatin (7.5 μM) treatment for another 2 h. The expression of the indicated proteins was detected by Western blot. GAPDH and Lamin B1 were detected as a loading control.

DNA-PK is a nuclear DNA-dependent protein kinase that is involved in cisplatin-induced DNA damage response^24^,^25^. We then investigated if DNA-PK is involved in cisplatin-induced cell death. The specific DNA-PK inhibitor NU7026 significantly increased cisplatin-induced cytotoxicity in both A549 and H2009 control cells (Fig. 5a,b), suggesting that DNA-PK plays a role in containing the cytotoxic effect of cisplatin. However, on the already high cytotoxicity background, the NU7026 had little effects on cisplatin-induced death in H4 KD cells (Fig. 5a,b), consistent with the weak cisplatin-induced DNA-PK activation in these cells (Fig. 5c). NU7026, which effectively inhibited cisplatin-induced DNA-PK activation, blocked the cisplatin-induced expression of RIP1, cIAP1, and XIAP in A549 control cells (Fig. 5d). Notably, NU7026 had no effect on H4 expression under the conditions with or without cisplatin treatment (Fig. 5d). It is worth noting that there was still a weak DNA-PK activation induced by cisplatin in H4 KD cells (Fig. 5c). However, this level of DNA-PK activation was unable to maintain expression of RIP1, cIAP1 and XIAP (Fig. 5e), suggesting that there is likely a threshold of DNA-PK activity for maintaining RIP1, cIAP1 and XIAP expression and cell survival. Nevertheless, these results strongly support that H4 modulates DNA-PK to retain expression of RIP1 and IAPs to attenuate cisplatin-induced cell death.

### H4 downregulation potentiates cisplatin’s antitumor activity *in vivo*

**Figure 6:**
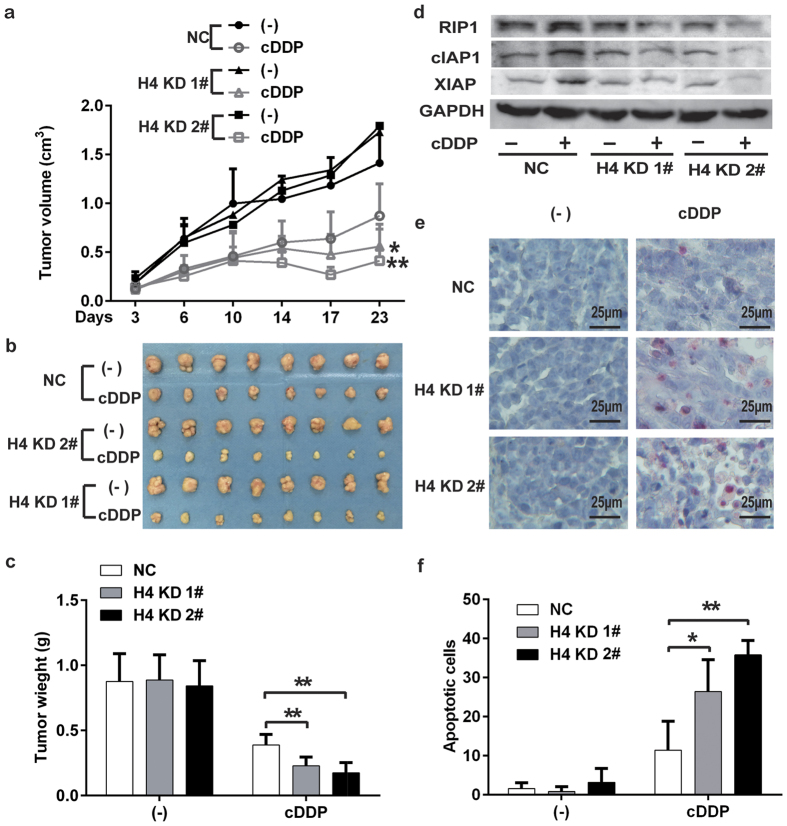
H4 knockdown enhances cisplatin’s antitumor activity *in vivo*. (**a**) A549-NC, A549-KD 1# or -KD 2#) cells (1 × 10^6^) were inoculated subcutaneously into the flanks of athymic nude mice. When the xenograft tumors were palpable, the mice were randomly divided into two groups receiving injection of cisplatin (i.p.) or PBS. The growth curve with the mean of tumor volume was shown. (**b**) Excised tumors. (**c**) Tumor weight. Columns, mean, bars, SD. (**d**) Expression of RIP1, cIAP1, and XIAP in tumor tissues was detected by Western blot. GAPDH was detected as a loading control. (**e**) Detection of apoptosis by TUNEL assay. Representative images were shown. (**f**) Apoptotic cells (TUNEL-positive cells) were counted in five fields (40×) from each group and the average of apoptotic cell numbers per field were shown as mean ± SD. *p < 0.05; **p < 0.01.

The role of H4 in the antitumor efficacy of cisplatin was investigated *in vivo* with a mouse xenograft tumor model. A549 control cells and H4 KD cell clones were injected subcutaneously into nude mouse for the development of xenograft tumors. Then the mice were treated with cisplatin. Consistent with the *in vitro* results, H4 KD significantly enhanced cisplatin’s antitumor activity (Fig. 6a–c). Downregulation of H4 was confirmed in the xenograft tumor tissues (Supplementary Fig. S5). Downregulation of RIP1 and IAPs after cisplatin treatment were detected in tumor tissues from H4 knockdown groups by Western blot (Fig. 6d). TUNEL assay was employed to examine whether the enhanced antitumor activity of cisplatin was because of potentiation of cisplatin-induced apoptosis in tumor tissues. As expected, the numbers of apoptotic cells in tumor tissues from the cisplatin-treated H4 KD groups were significantly increased compared to that in tumor tissues from the cisplatin-treated control group (Fig. 6e,f). Thus, it is validated that H4 downregulation is able to potentiate cisplatin’s antitumor activity *in vivo*.

## Discussion

In the present study, we found that histone H4 expression plays an important role in cisplatin-induced apoptotic cytotoxicity in cancer cells. This novel finding highlights that, in addition to histone modification, regulating the expression level of certain histone subtype is another chemoresponse mechanism in cancer cells. We show that the NF-κB and Akt signaling pathways cooperatively maintain the expression level of H4, which suppresses RIP1 degradation, ROS accumulation and IAPs degradation to attenuate cisplatin-induced cytotoxicity. Further, with a DNA-PK inhibitor, we found that histone H4 maintains DNA-PK activation to retain expression of RIP1 and IAPs in cisplatin-treated cells. These results establish a novel mechanism by which NF-κB and Akt contribute to chemoresistance involving a signaling pathway consisting of histone H4, DNA-PK, RIP1 and IAPs that attenuates ROS-mediated apoptosis, and targeting this pathway may improve anticancer efficacy of platinum-based chemotherapy.

Extended from our previously studies showing that concurrent blockage of the NF-κB and Akt signaling pathways sensitized cancer cells to cisplatin-induced cell death, we investigated cisplatin response regulation mechanism and found that all histone H4- coding genes were down-regulated in NF-κB- and Akt-blocked cells. Knockdown of histone H4 expression significantly increased sensitivity to cisplatin, suggesting that H4 is a molecule co-targeted by NF-κB and Akt for attenuating cisplatin cytotoxicity. Histone H4, similar to other histones, is encoded by multiple genes located to different loci and there is no sequence variant of these genes^14^, suggesting that there is an evolutionary advantage in keeping multiple copies for maintaining H4 abundance for maintaining normal cell function and stress response. The expression of histone genes is controlled at transcriptional and post-transcriptional levels^14^, and the latter consists of RNA processing and translation, and RNA stability^26^. Also, histone expression levels are regulated by phosphorylation and ubiquitylation-dependent proteolysis^27^. While NF-κB is a transcription activator and Akt regulates gene expression through protein phosphorylation, how these two pathways regulate H4 expression is not understood, which would be an interesting focus of future studies.

Accumulating reports have shown that modifications of histones such as acetylation, phosphorylation and methylation play an important role in regulating gene expression as well as chemosensitivity. However, there are only few reports on the role of histones expression level in chemosensitivity^17^,^19^. In budding yeast, artificial histone overexpression resulted in enhanced DNA damage sensitivity whereas deletion of a H3–H4 gene pair reduced the levels of free H3 and H4 and resulted in resistance to DNA damaging agents^28^. These findings imply that to retain a proper expression level of histone is important for proper DNA damage response. Here we for the first time found that inhibiting histone H4 expression sensitized cancer cells to cisplatin-induced cell death. Notably, H4 knockdown in our system had little effect on cell proliferation, suggesting that the H4 expression level sufficient for cell proliferation may not fully support cisplatin-induced DNA damage response. How reduced H4 expression impact DNA damage response is currently unknown. It is interesting to investigate if reduced H4 protein level results in reduced nucleosomes, leading to structurally loose chromatin, and increase DNA accessibility by cisplatin, alike that of histone acetylation.

We further found that cisplatin caused significant reduction in the expression of RIP1, cIAP1 and XIAP in H4 KD cells, and determined a signaling cascade consisting of RIP1/ROS/IAPs for regulating cisplatin cytotoxicity, which is consistent with our previous studies showing that RIP1 suppresses cisplatin-induced ROS accumulation to retain expression of IAPs^23^. The reduction of RIP1 and IAPs was mediated by proteasomal degradation but not the result of apoptosis. Importantly, the decrease of the expression of RIP1, cIAP1 and XIAP in H4 KD cells occurred as early as 30 min after cisplatin treatment, suggesting the regulation of these proteins was an early event that plays an important role in regulating cisplatin-induced cytotoxicity. All these results suggest RIP1 reduction contributes to enhancement of cisplatin-induced ROS accumulation, which in turn, leads to degradation of IAPs in H4 KD cells.

DNA-PK, a key component of the nonhomologous end-joining (NHEJ) pathway of double-strand break (DSB) repair^29^,^30^,^31^,^32^,^33^,^34^, is a molecular sensor for DNA damage^35^,^36^,^37^,^38^,^39^,^40^. It has been reported that compromised DNA-PKcs expression and activity results in decreased DSB repair and increased radiosensitivity^29^,^30^,^31^,^32^,^33^,^34^. In this study, we found that cisplatin-induced DNA-PK activation was inhibited in H4 KD cells and inhibiting DNA-PK activity significantly increased cisplatin sensitivity in cancer cells. Suppressing DNA-PK with NU7026 blocked the cisplatin-induced expression of RIP1, cIAP1, and XIAP in A549 control cells, which was associated with increased cisplatin cytotoxicity. It should be noted that although NU7026 is highly selective towards DNA-PK, the possibility that other PI3K-related kinases such as ATM and ATR are involved could not be completely excluded. While further investigation on this point is needed, our results suggest that H4 modulates DNA-PK to retain expression of RIP1 and IAPs that attenuates cisplatin-induced cell death.

In conclusion, our study establishes a novel mechanism by which NF-κB and Akt contribute to chemoresistance that through maintaining histone H4 expression for activating the DNA-PK/RIP1/IAPs signaling pathway that attenuates ROS-mediated apoptosis. Targeting this pathway may be exploited for improving the anticancer efficacy of platinum-based chemotherapy.

## Materials and Methods

### Reagents

Anti-phospho-DNA-PKcs and -histone H4 antibodies were from Abcam (Cambridge, MA, UN). Anti-DNA-PKcs antibody was the product of BBI Life Science (Shanghai, China). Anti-IKKβ antibody was from Merck Millipore (Billerica, MA, USA). Anti-Akt1 and -XIAP antibodies were purchased from Cell Signaling Technology (Danvers, MA, USA). Anti-RIP1 and -PARP antibodies were obtained from BD Bioscience (San Diego, CA, USA). Anti-active caspase 3 and -cIAP1 antibodies were the products of R&D (Minneapolis, MN, USA). Cisplatin, puromycin, butylated hydroxyanisole (BHA), N-acetyl-L-cysteine (NAC), and Chloroquine (CQ) were purchased from Sigma-Aldrich (St. Louis, MO, USA). The pan-caspase inhibitor zVAD and DNA-PK inhibitor NU7026 were the products of Merck Millipore (Billerica, MA, USA). Small interfering RNA (siRNA) targeting different proteins and negative control siRNA were from Guangzhou RiboBio Co. (Guangzhou, China). siRNA transfection reagent was from Polyplus transfection (Illkirch, France). TRIzol reagent was from Invitrogen (Carlsbad, CA, USA).

### Cell culture and cell death assay

Human lung adenocarcinoma cells A549 and H2009 were cultured in PRMI 1640 medium (Carlsbad, CA, USA) supplemented with 10% fetal bovine serum (Carlsbad, CA, USA) and 100 U/ml of penicillin-streptomycin. The cells were incubated at 37 °C in a humidified atmosphere of 5% CO_2_. Cell death was detected by a cytotoxicity assay based on the release of lactate dehydrogenase (LDH) using a cytotoxicity detection kit (Promega, Madison, WI, USA) as described previously^41^. All the experiments were repeated three to five times and the average is shown in each figure.

### RNA interference

Cells were transfected with small interfering RNA targeting Akt1, IKKβ, or histone H4, and negative control siRNA by using siRNA transfection reagent following the manufacturer’s instructions (Illkirch, France). Forty-eight hours after transfection, the inhibitory effect of siRNA on proteins expression was detected by quantitative real-time RT‐PCR and Western blot. To evaluate the effect on cisplatin sensitivity, the siRNA transfected cells were treated with cisplatin for another 72 h, and cell death was measured by LDH release-based cell death assay.

### Lentivirus infection and establishment of stable cell lines

The psi-LVRH1GP shRNA lentiviral vector with shRNA against histone H4, which targets most H4 genes, and control vectors were purchased from GeneCopoeia (Guangzhou, China). The targeting sequences are: CTGCCATGGATGTGGTTTA and GAATTTCCGGTTTGATTTA. Viruses were produced and packaged in HEK293T cells using Lenti-Pac™ HIV Expression Packaging Kit following the instructions of the manufacturer (GeneCopoeia, Guangzhou, China). The shRNA viral vector targeting histone H4 was used to establish histone H4 knockdown cell sublines. The vector harboring control scramble shRNA was used as a negative control. Cells were infected with viruses and selected with 5 μg/ml of puromycin. Positive cell clones were expanded and maintained in a medium supplemented with 1 μg/ml puromycin.

### Quantitative real-time RT-PCR

Total RNA was extracted with TRIzol reagent and reverse transcribed to cDNA using a reverse transcription kit (Thermo Scientific, MA, USA). Quantitative real-time PCR was carried out with SYBR master mixture on Eppendorf Connect Real-Time PCR platform following the manufacturer’s instructions (Thermo Scientific, MA, USA). The primers used were as follows: histone H4, 5′-AAAGTGCTGCGGGATAACAT-3′ (forward) and 5′-CCTTGAGAACGCCACGAGT-3′ (reverse); histone H2AB, 5′-ATAAACTCTTGGGGCGTGTG-3′ (forward) and 5′-TTGGCCTTATGATGGCTCTC-3′; GAPDH, 5′-AGCCACATCGCTCAGACAC-3′ (forward) and 5′- GCCCAATACGACCAAATCC -3′ (reverse). Data were analyzed as Relative Quantitation (RQ) with respect to a calibrator sample using the 2^−ΔΔCt^ method. The results were presented as mean ± SD from three experiments.

### Western blot

Whole cell extracts were prepared by lysing the cells in M2 buffer [20 mM Tris-HCl (pH 7.6), 0.5% NP-40, 250 mM NaCl, 3 mM EDTA, 3 mM EGTA, 2 mM DTT, 0.5 mM phenylmethylsulfonyl fluoride, 20 mM β-glycerophosphate, 1 mM sodium vanadate, and 1 μg/ml leupeptin]. Total protein extracts from tumor xenograft tissues were prepared by smashing tumor tissues into smaller pieces, adding M2 lysis buffer, homogenized on ice, and then incubated on ice for 30 min. For the detecting histone H4 protein, cell extracts were prepared by lysing the cells with Total Histone Extraction Kit following the instructions of the manufacturer (Epigentek, NY, USA). Then, equal amounts of total protein were resolved by SDS-PAGE and the proteins of interest were probed by Western blot and visualized by enhanced chemiluminescence according manufacturer’s instructions (Millipore, Billerica, MA, USA) in the Gel Imaging System (Bio-Rad, Hercules, CA, USA). The results were analyzed with the Quantity One software (Bio-Rad, Hercules, CA, USA) to determine the relative ratio. The value of control was set as 1. The uncropped membranes are shown in Supplementary Fig. S6.

### Detection of ROS

Cells cultured in 12-well plates were treated with cisplatin as indicated in each figure legend. Cells were then stained for 30 minutes with 5 μM of CellROX^®^ Deep Red reagent (Thermo Fisher Scientific, MA, USA), which has an Excitation/Emission wavelength of 640/665 nm. The stained cells were washed 3 times with PBS, and cellular fluorescence was measured with the Varioskan Flash Multimode Reader (Thermo Fisher Scientific, MA, USA).

### *In vivo* xenograft tumor model and chemotherapy study

All procedures involving animals and their care were conducted in accordance with the guidelines of, and were approved by, the Institutional Animal Care and Use Committee of Sichuan University. Athymic male nude mice (6 weeks old) were randomly divided into three groups and inoculated subcutaneously into the flanks with A549 control cells (A549-NC) or H4 knockdown cells (A549-KD 1# or A549-KD 2#) (1 × 10^6^ cells), respectively. When the xenograft tumors were palpable, the mice were randomly divided into two groups and administrated with the following agents by i.p. injection for a total of 7 injections in 26 days: (a) PBS control; (b) 4.5 mg/kg of cisplatin. The length (*l*) and width (*w*) of tumor were measured using a micrometer caliper and tumor volume (V) was calculated using the following formula: *V = lw*^2^/2. At the end of experiment, animals were euthanized. Excised tumors were measured and weighed^42^. The tumor tissue was fixed in 4% paraformaldehyde for subsequent TUNEL assay and the remainder was snap-frozen in liquid nitrogen for Western blot detections.

### TUNEL assay

The TUNEL assay based on labeling of DNA strand breaks was applied to detect apoptosis in tumor tissues with the *In Situ* Cell Death Detection Kit following the manufacturer’s instructions (Roche Applied Science, Mannheim, Germany). Briefly, the tissue sections were dewaxed, rehydrated, treated with protease K and permeabilized, followed by incubation with the terminal deoxynucleotidyl transferase labeling reaction mixture for 60 min at 37 °C. The slides were then incubated with anti-fluorescein antibody conjugated with alkaline phosphatase for 30 min at 37 °C. Finally, AP-red and haematoxylin were added for color development^43^. TUNEL-positive cells were counted in five fields (40×) and average number of per field was shown. Images were taken by using the Image-Pro Express software (Media Cybernetics, USA).

### Statistical analysis

All numerical data are presented as mean ± standard deviation (SD). Statistical significance was analyzed by paired Student’s *t* test using SPSS statistics software package and *p* < 0.05 was used for significance.

## Additional Information

**How to cite this article**: Wang, R. *et al*. Histone H4 expression is cooperatively maintained by IKKβ and Akt1 which attenuates cisplatin-induced apoptosis through the DNA-PK/RIP1/IAPs signaling cascade. *Sci. Rep.* **7**, 41715; doi: 10.1038/srep41715 (2017).

**Publisher's note:** Springer Nature remains neutral with regard to jurisdictional claims in published maps and institutional affiliations.

## Supplementary Material

Supplementary Methods,Table and Figures
