# Supplementary material for: Histone H4 expression is cooperatively maintained by IKKβ and Akt1 which attenuates cisplatin-induced apoptosis through the DNA-PK/RIP1/IAPs signaling cascade
Source: Sci Rep. 2017 Jan 31;7:41715. doi: 10.1038/srep41715 (PMC5282510; doi:10.1038/srep41715)

Histone H4 expression is cooperatively maintained by IKKβ and Akt1 which attenuates cisplatin-induced apoptosis through the DNA-PK/RIP1/IAPs signaling cascade

Ruixue Wang[a], Xuelian Zheng[a], Lei Zhang[a], Bin Zhou[a], Huaizhong Hu[a], Zhiping Li[b], Lin Zhang[a, d], Yong Lin[a, c] *, Xia Wang[a, d]*

[a] Laboratory of Molecular and Translational Medicine, Key Laboratory of Birth Defects and Related Diseases of Women and Children (Sichuan University) of Ministry of Education, Department of Obstetrics and Gynecology, West China Second University Hospital, Sichuan University, Chengdu 610041, China; [b]Department of Abdominal Oncology, Cancer Center, West China Hospital, Sichuan University, Chengdu 610041, China; [c]Molecular Biology and Lung Cancer Program, Lovelace Respiratory Research Institute, 2425 Ridgecrest Dr., SE., Albuquerque NM 87108, USA; [d]Department of Immunology, West China School of Preclinical and Forensic Medicine, Sichuan University, Chengdu 610041, China.

* Corresponding authors: Xia Wang, Laboratory of Molecular and Translational Medicine, West China Second University Hospital, Sichuan University, Chengdu 610041, China, Tel: +86-28-85503773; Fax: +86-28-85503604; E-mail: xiawang@scu.edu.cn. Yong Lin, Molecular Biology and Lung Cancer Program, Lovelace Respiratory Research Institute, Albuquerque, NM87108, USA. Tel: 505-348-9645; Fax:505-348-8567; Email: ylin@lrri.org.

Supplementary methods

Gene expression array assays

Two hundred nanograms of total RNA from each sample were used to generate amplified and biotinylated sense-strand cDNA from the entire expressed genome according to the GeneChip WT PLUS Reagent Kit User Manual (P/N 703174, Affymetrix Inc., Santa Clara, CA). The GeneChip® Human Gene 1.0 ST Arrays were hybridized for 16 hours in a 45°C incubator (Affymetrix GeneChipHybridization Oven 640), rotated at 60 rpm. After hybridization, the microarrays were washed and stained using the Fluidics Station 450 followed by scanning with the Affymetrix GeneChip Scanner 3000 7G according to manufacturer's instructions. The raw data were normalized with the free software Expression Console 1.4 provided by Affymetrix using the quantile normalization of robust multiarray average (RMA) method. The GeneChip® Human Gene 1.0 ST Array analysis was conducted by CapitalBio Technology (Beijing, China).

Cell proliferation assay

For cell proliferation assay, cells were cultured overnight in serum-free RPMI 1640 medium. The medium was then replaced with RPMI 1640 supplemented with 10% fetal bovine serum (FBS). At different time points after incubation (1, 2, 3, or 4 days), CCK8 solution was added to each well and incubated at 37°C for 1 h. The absorbance was read at 450 nm using a plate reader. All the experiments were repeated three to five times and the average is shown.

Cell cycle analysis by flow cytometry

Flow cytometry was used to analyze cell cycle distribution. After overnight culture in serum-free medium, the cells were cultured in full medium containing 10% FBS with or without cisplatin for the times as indicated in figure legend. Cells were then trypsinized, washed twice with PBS and fixed in 70% ethanol on ice for 30 min. The fixed cells were stained with far-red fluorescent DNA dye DRAQ5™, 1, 5– bis{[2-(di- methylamino) ethyl] amino}- 4, 8-dihydroxyanthracene-9, 10-dione at room temperature for 30 min. The stained cells were analyzed with the FL4 channel (675 nm) using a FAScan flow cytometer (Beckman coulter Cytomics FC 500

flow cytometer). The percentages of cells in the G0/G1, S, and G2/M phases of cell cycle were determined by DNA content.

Fluorescence Microscopy

H4 knockdown cells (A549-KD 1#) were transfected overnight with pR3RED along with pcDNA, or RIP1-, cIAP1- or XIAP- expression plasmids using X-tremeGENE HP DNA Transfection Reagent (Roche, Mannheim, Germany). Forty-eight hours after transfection, the cells were treated with cisplatin for another 48 h. Images were captured under an Olympus CX31 microscope and an Olympus DP72 camera with Instudio software. The representative images of three experiments were shown. The percentage of survival fluorescent cells was calculated.

Supplementary Table 1: Reduced histone H4-coding gene expression in Akt1-, IKKβ- or Akt1 and IKKβ-knockdown A549 cells

Gene symbol mRNA Accession	Negative Control	Akt1 KD		IKKβ KD		Akt1 and IKKβ KD	
	Reads	Reads	Fold*	Reads	Fold*	Reads	Fold*
HIST1H4B NM_003544	1287	1142	0.89	930	0.72	616	0.48
HIST1H4C NM_003542	2279	2006	0.88	1679	0.74	1150	0.50
HIST1H4K NM_003541	857	798	0.93	674	0.79	450	0.53
HIST1H4K NM_021968	433	390	0.90	337	0.78	235	0.54
HIST1H4H NM_003543	173	138	0.80	144	0.83	97	0.56
HIST1H4A NM_003538	232	201	0.87	169	0.73	135	0.58
HIST1H4D NM_003539	325	261	0.80	237	0.73	189	0.58
HIST2H4B NM_001034077.4	1062	974	0.92	922	0.87	614	0.58
HIST1H4E NM_003545	310	306	0.98	270	0.87	190	0.61
HIST4H4 NM_175054	32	23	0.71	32	1.00	21	0.65
HIST1H4I NM_003495	183	166	0.91	151	0.83	121	0.66
HIST1H4L NM_003546	19	18	0.95	16	0.84	14	0.74
HIST2H4A NM_003548	785	788	1.00	795	1.01	584	0.74
HIST1H4F NM_003540	28	21	0.75	23	0.82	25	0.89

*Fold change was calculated as the ratio of the reading value of respective stable gene expression knockdown cells over that of Negative Control cells.

Supplemental figures with legends

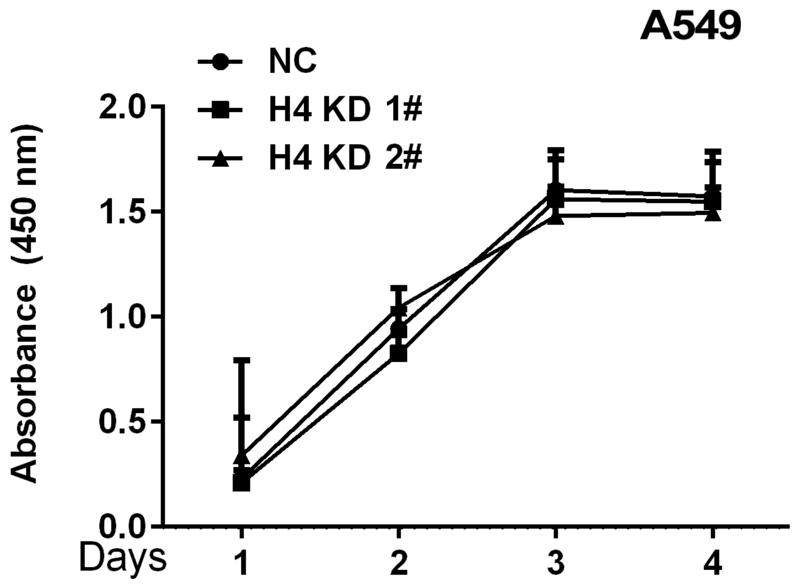

Fig. S1. Cell proliferation was measured by using a CCK-8 kit. A549 NC cells and H4 KD cell clones (H4 KD 1# and KD 2#) were cultured overnight in serum-free RPMI 1640 medium. The medium was then replaced with RPMI 1640 supplemented with 10% fetal bovine serum. At different time points after incubation (1, 2, 3, or 4 days), CCK8 solution was added to each well and incubated at 37°C for 1 h. The absorbance was then read at 450 nm using a plate reader. All the experiments were repeated three to five times and the average is shown.

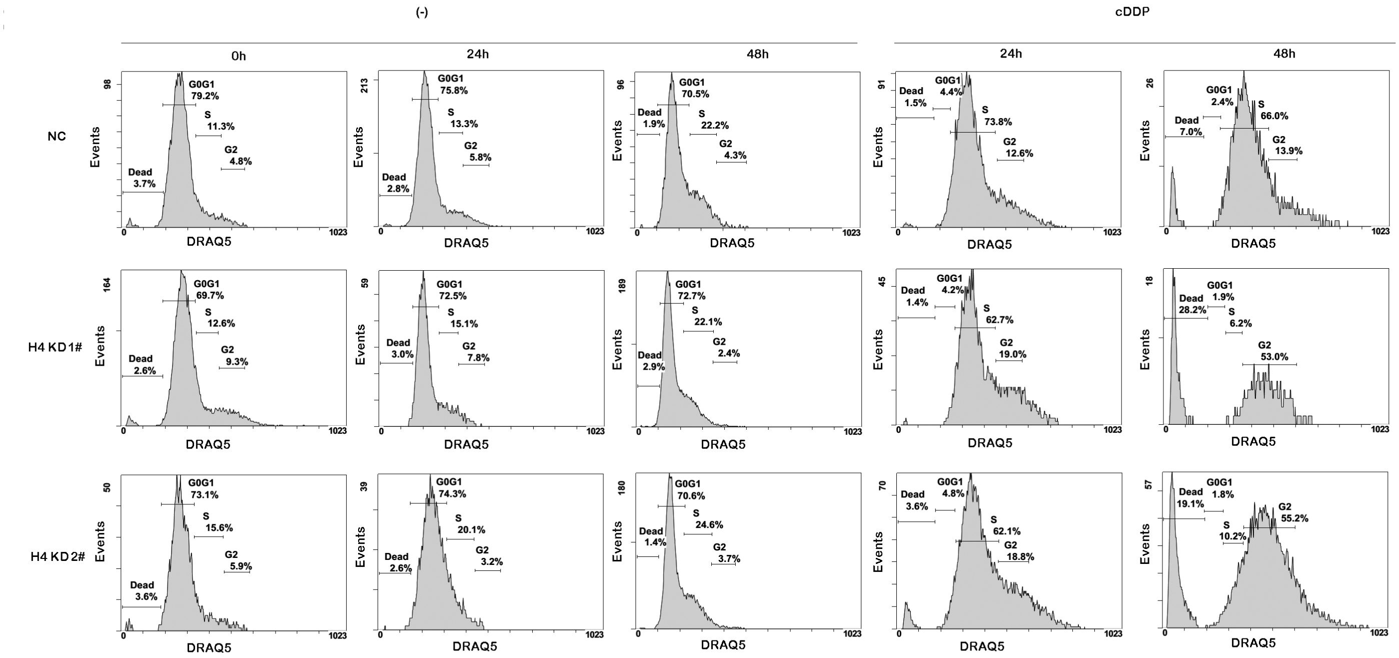

Fig. S2. Cell cycle distribution was measured by flow cytometry. After overnight culture in serum-free medium, the cells were cultured in full medium with or without cisplatin (7.5 μM) for indicated the times, trypsinized, washed twice with PBS and fixed in 70% ethanol on ice for 30 min. The fixed cells were stained with the far-red fluorescent DNA dye DRAQ5™ (20 μM) at room temperature for 30 min, and analyzed using a FAScan flow cytometer (channel FL4, excitation: 675 nm, Beckman coulter Cytomics FC500 5 color flow cytometer). The percentage of cells in the G0/G1, S, and G2/M phases of cell cycle were determined by cellular DNA content.

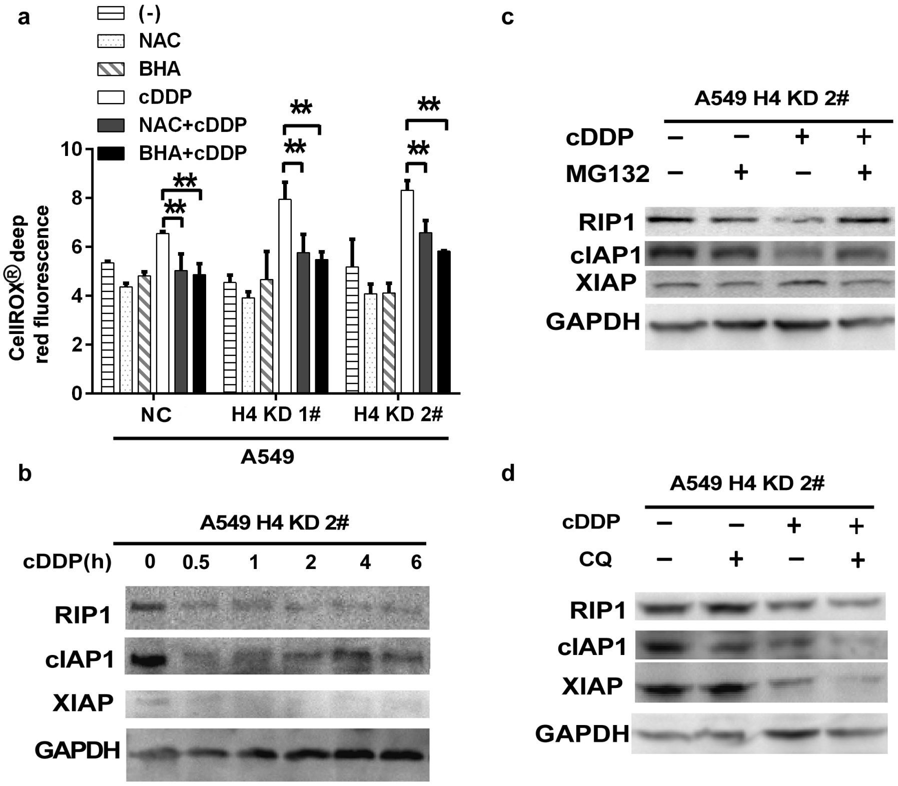

Fig. S3. (a) Negative control (NC) and H4 knockdown A549 cell clones were pre-treated with NAC (1 mM) or BHA (100 μM) for 1 h. Then the cells were treated with cisplatin (7.5 μM) for 7 h followed by incubating with CellRox dye for 30 min. Then the cells were collected and cellular fluorescence was measured with the Varioskan Flash Multimode Reader. Columns, mean of three experiments; bars, SD. ***P*<0.01. (b) H4 knockdown cell clone (A549-KD 2#) were treated with cisplatin (7.5 μM) for indicate times. The expression of RIP1, cIAP1, and XIAP were detected by Western blot. GAPDH was detected as a loading control. (c) and (d) H4 KD 2# cells were pretreated with MG132 (10 μM), or chloroquine (CQ, 20μM) for 1h. Then the cells were treated with cisplatin (7.5 μM) for another 2 h. The expression of RIP1, cIAP1, and XIAP were detected by Western blot. GAPDH was detected as a loading control.

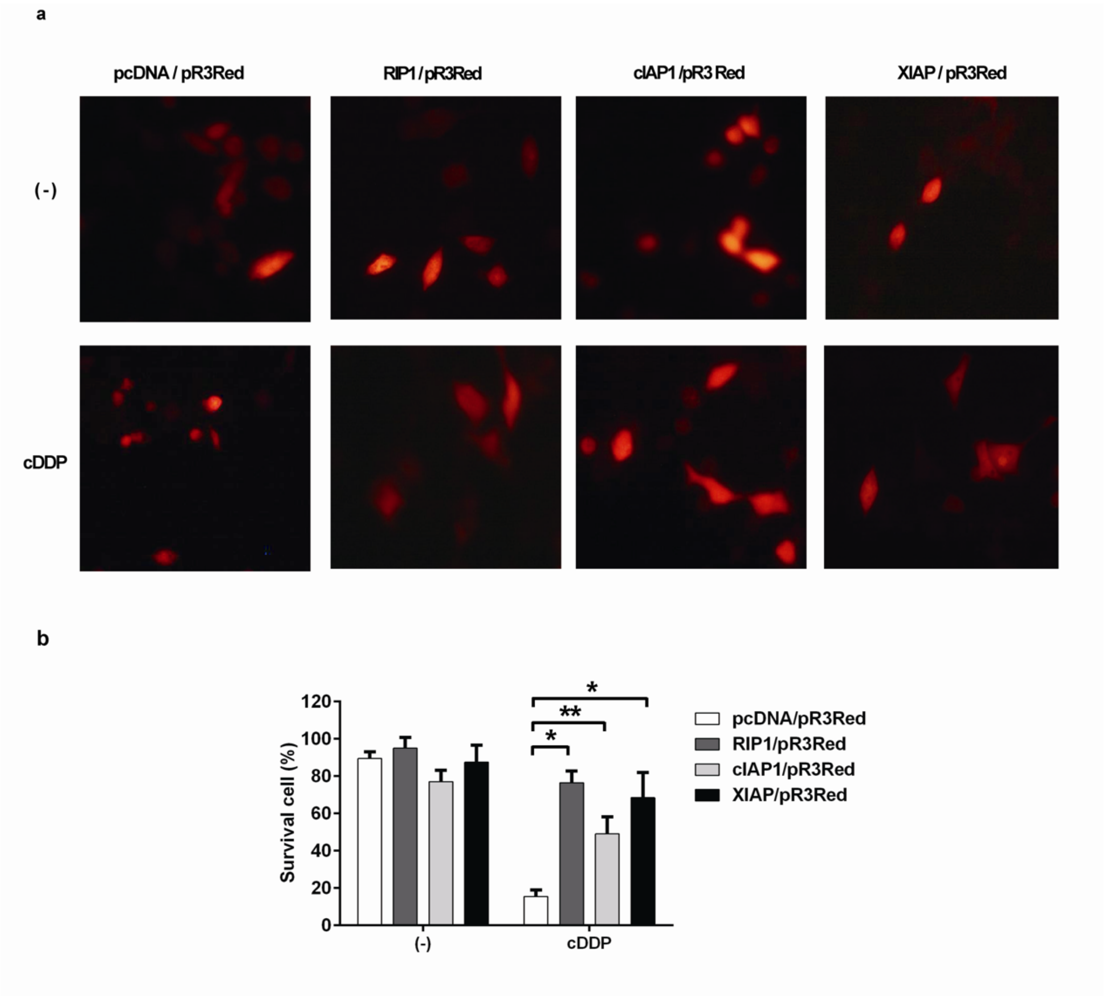

Fig. S4. Ectopic expression of RIP1 and IAPs protects H4 knockdown A549 cells against cisplatin-induced cytotoxicity. (a) After co-transfection with pR3RED and pcDNA, RIP1, cIAP1, or XIAP expression plasmids, the H4 knockdown cells (A549-KD 1#), were exposed to cisplatin for 48 h. The photographs were taken under a fluorescence microscope. The empty vector pcDNA was used as a negative control. (b) Survival cells positive for pR3RED was quantified. Data are presented as the means ± SD of 3 independent experiments performed in triplicate. **P<0.01.

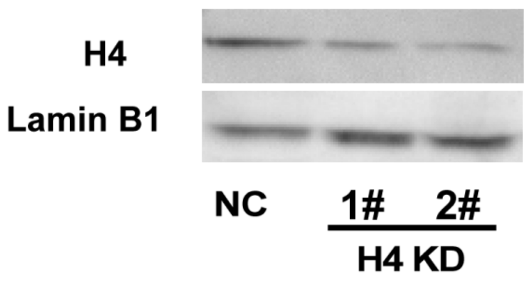

Fig. S5. H4 protein expression in representative tumor from each group was detected by Western blot. Lamin B1 was measured as an input control.

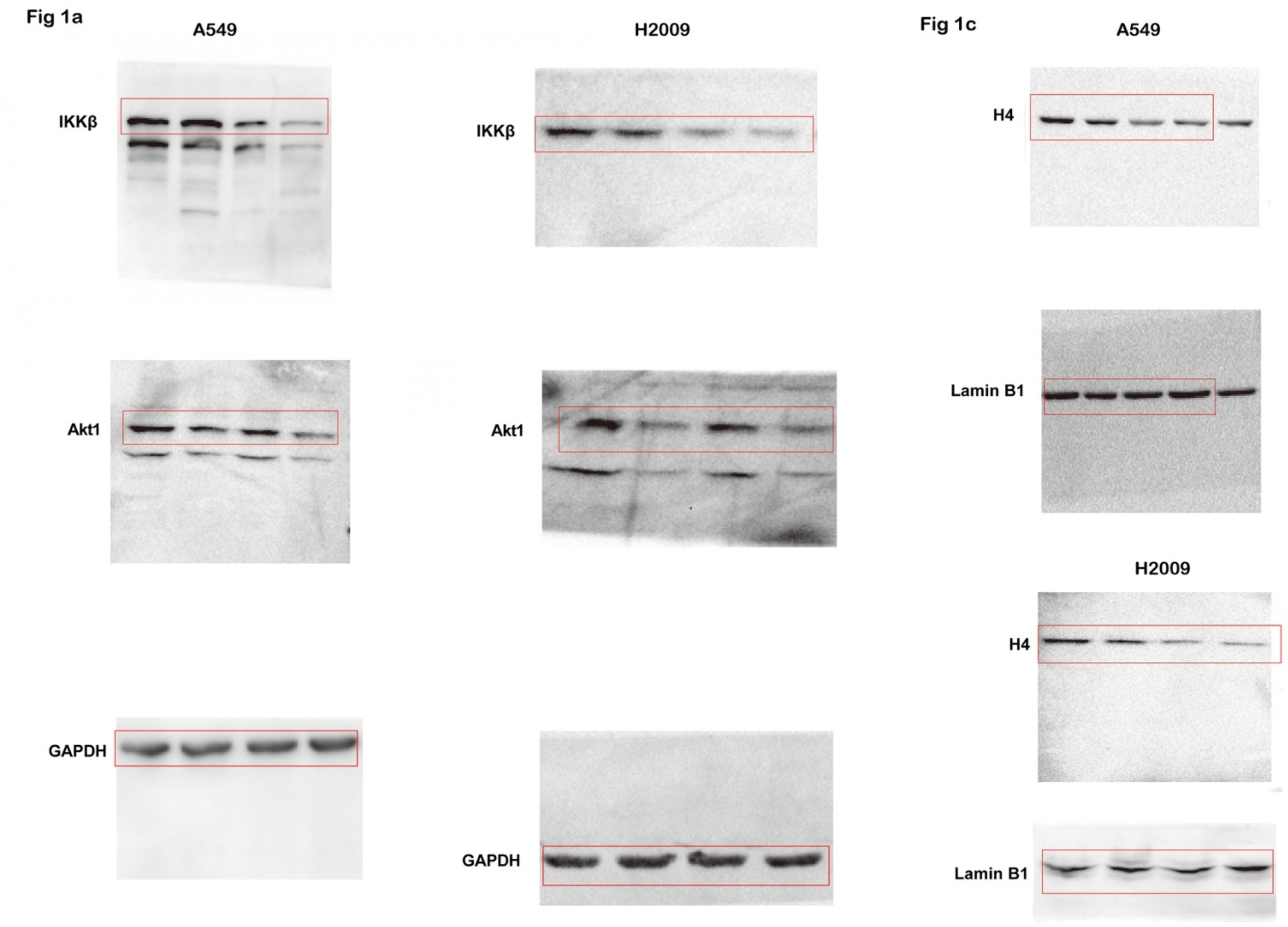

Fig S6. Images of full blots that were cropped for presentation are shown. Red rectangles indicate regions used in figures.

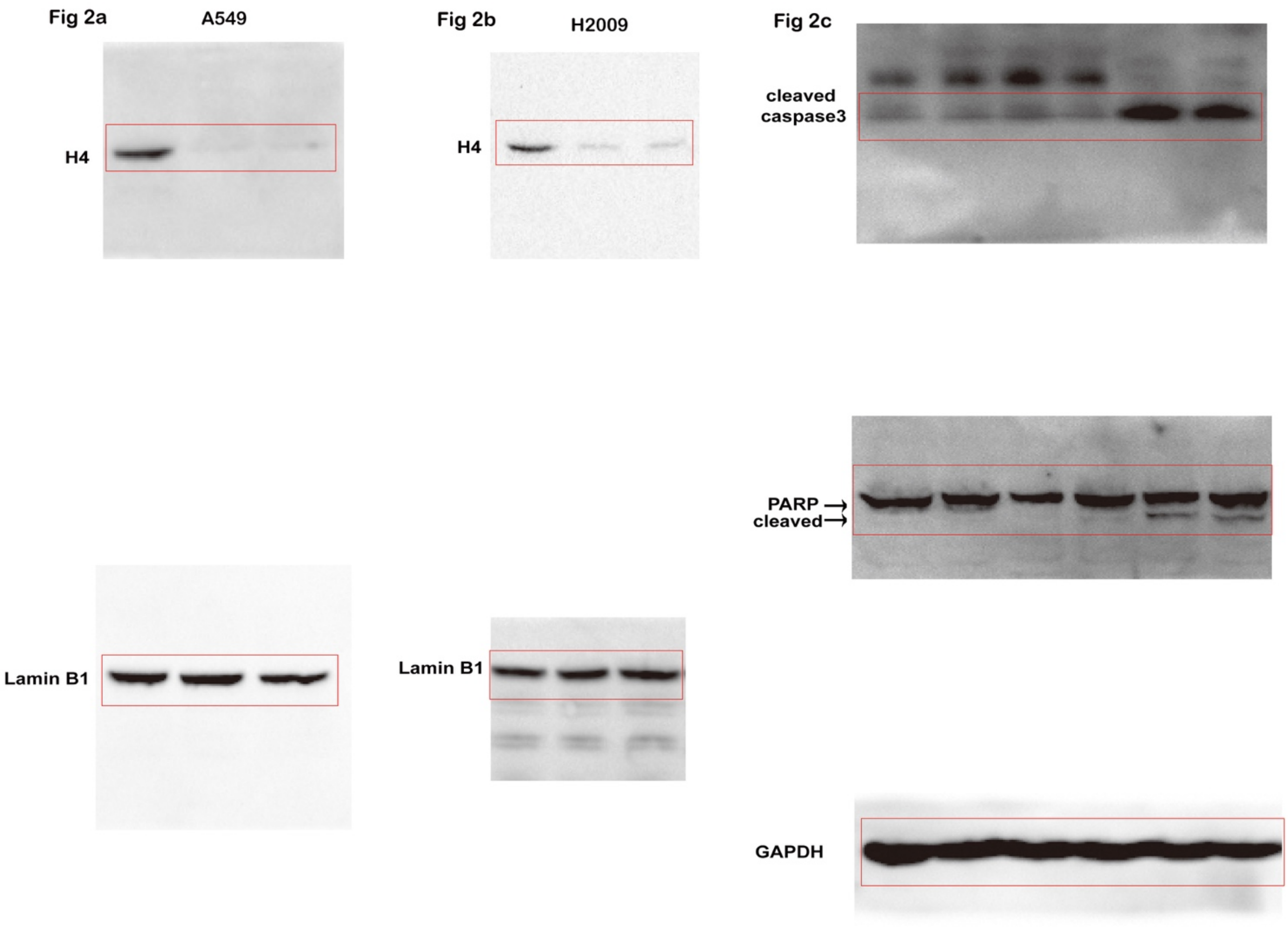

Fig S6 continued

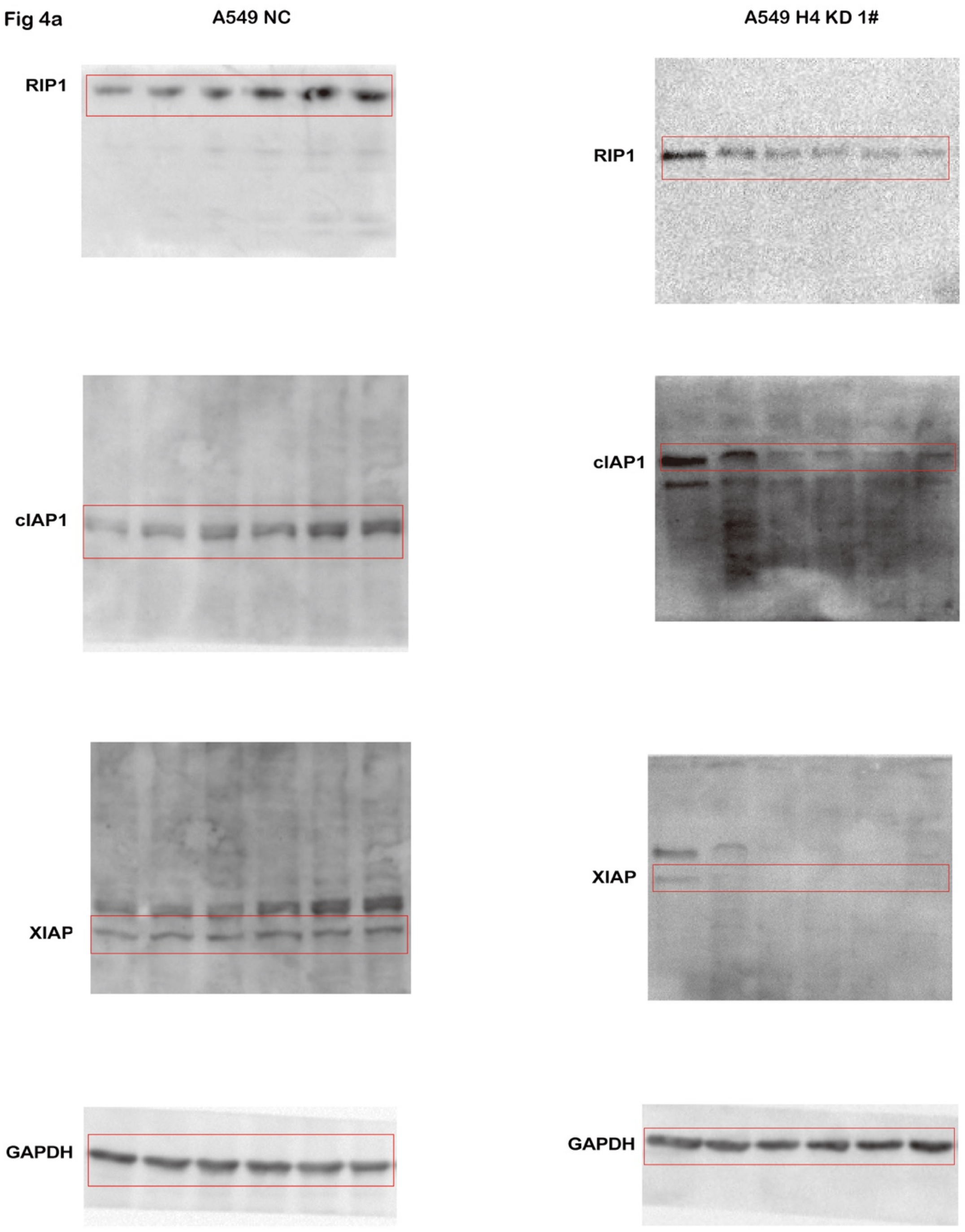

Fig S6 continued

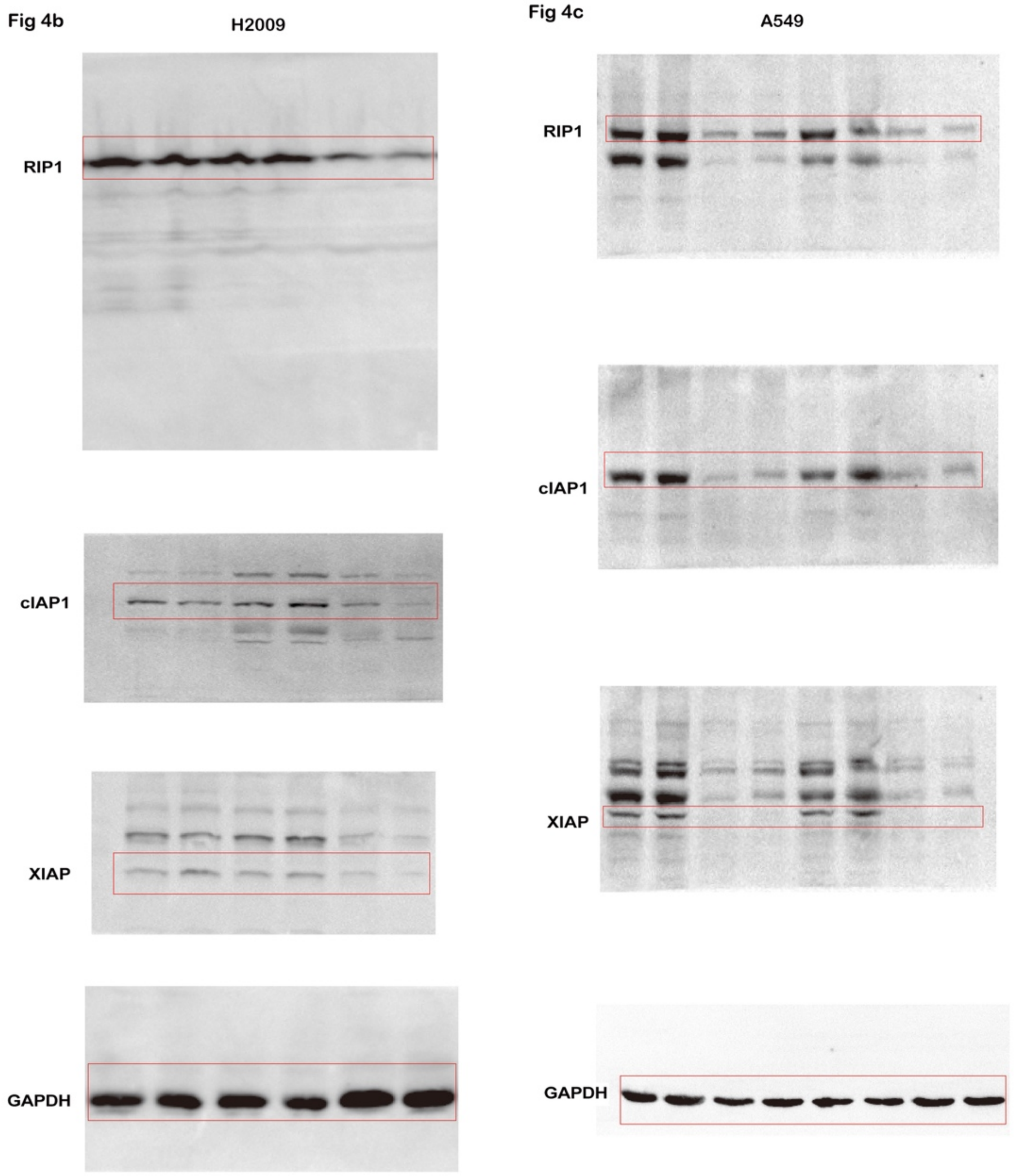

Fig S6 continued

Fig 4d A549 H4 KD 1#

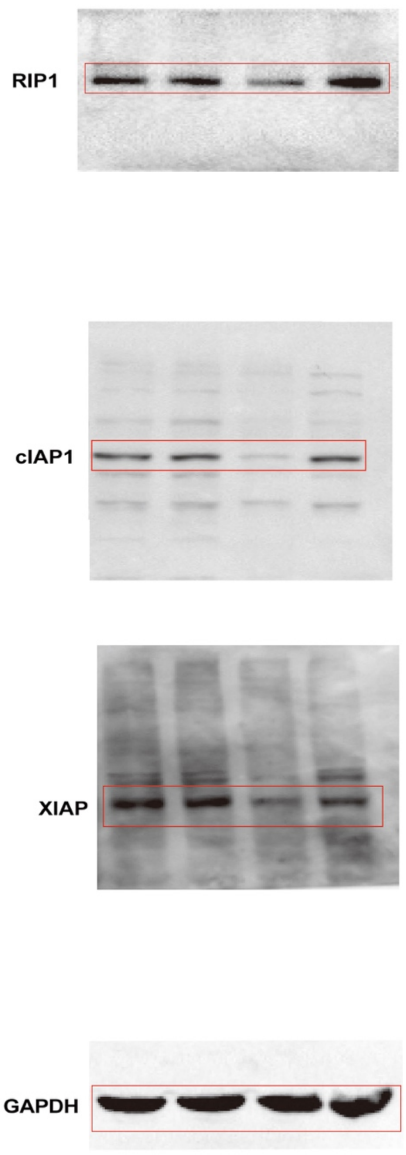

Fig 4e A549 H4 KD 1#

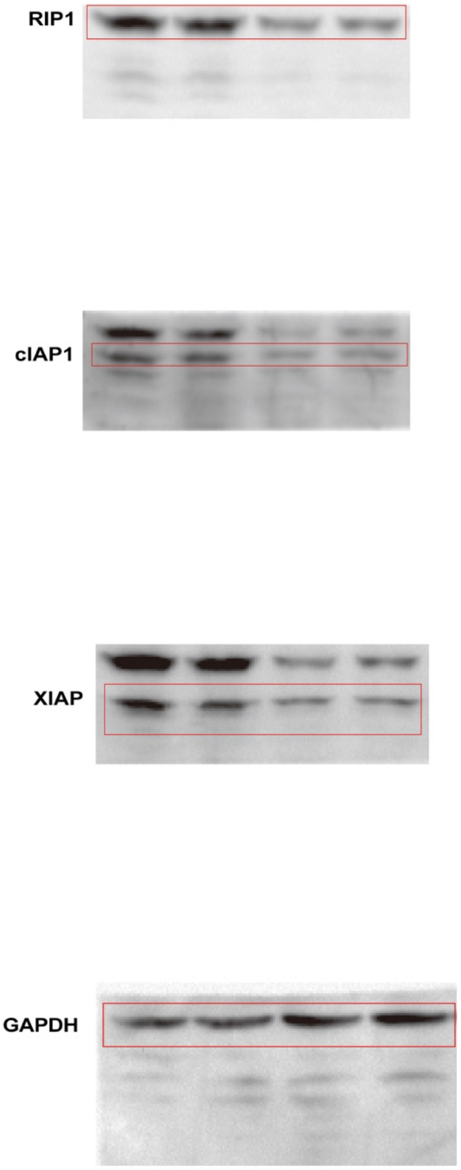

Fig S6 continued

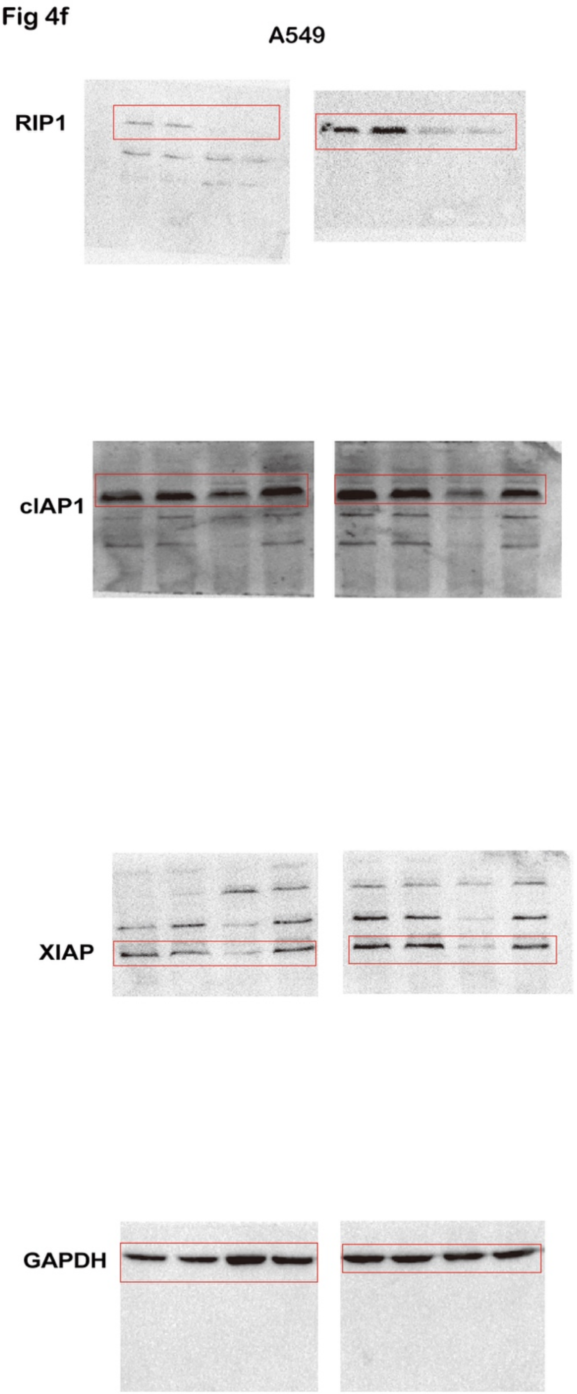

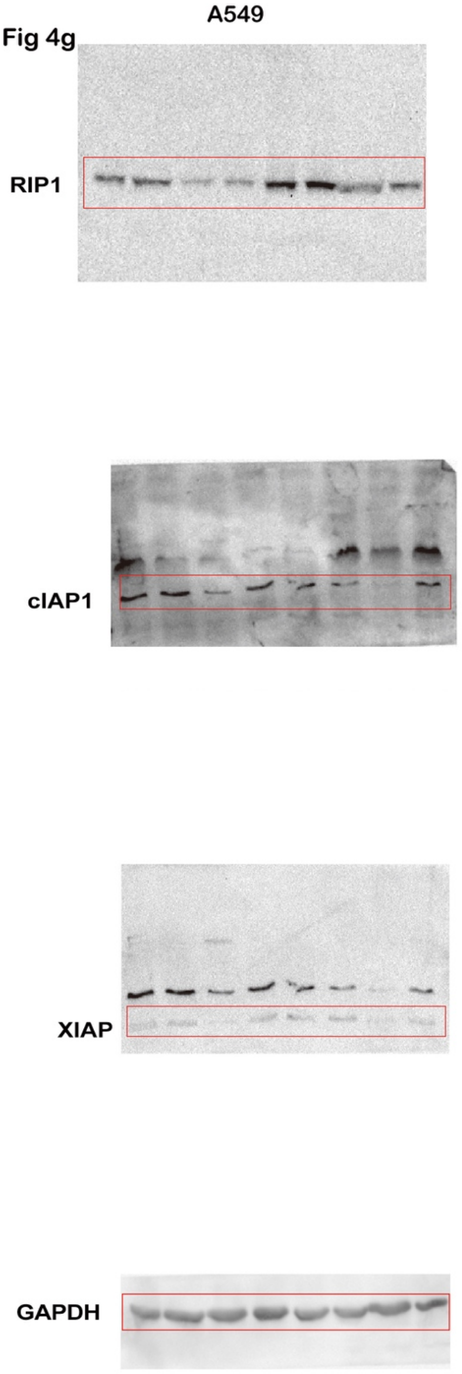

Fig S6 continued

Fig 5c

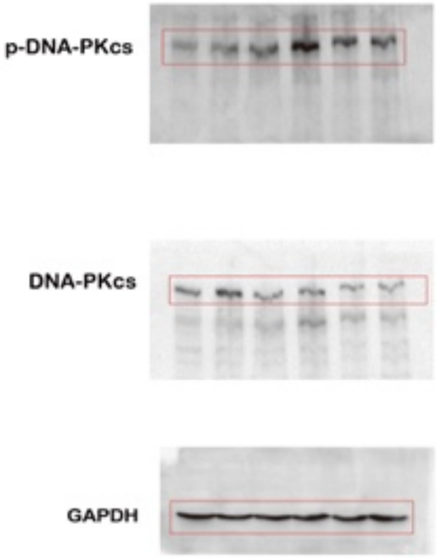

Fig 5d

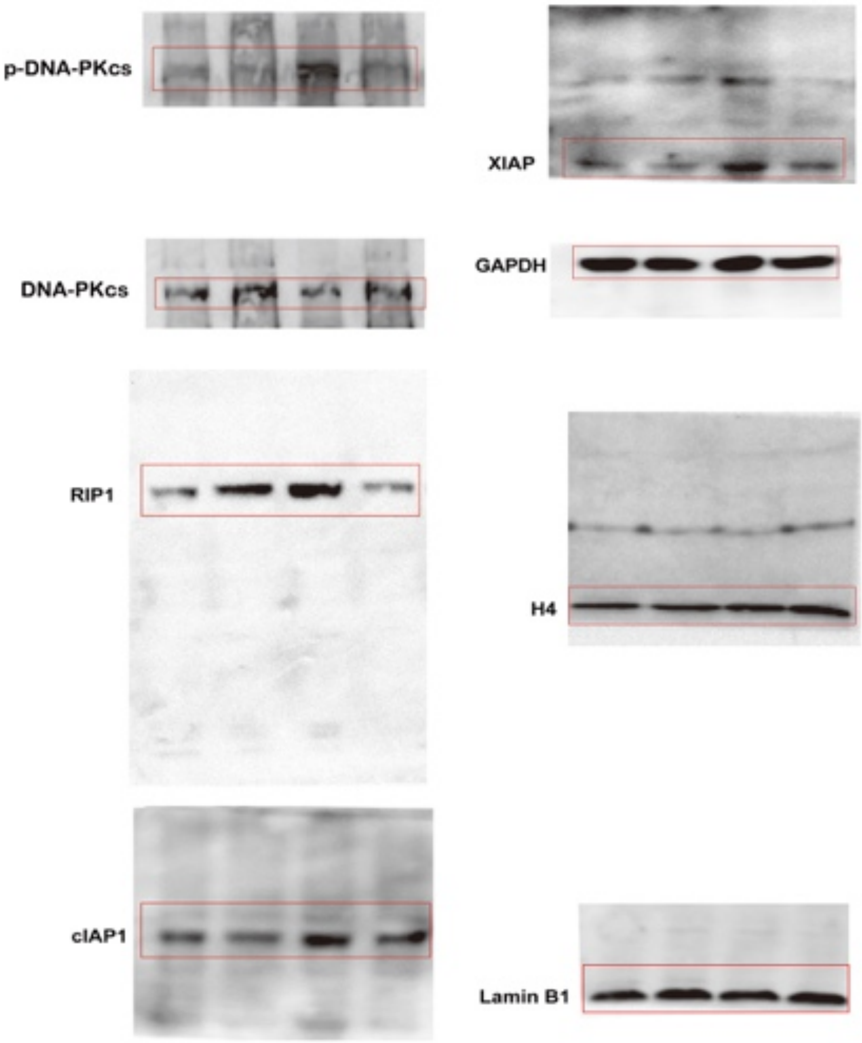

Fig S6 continued

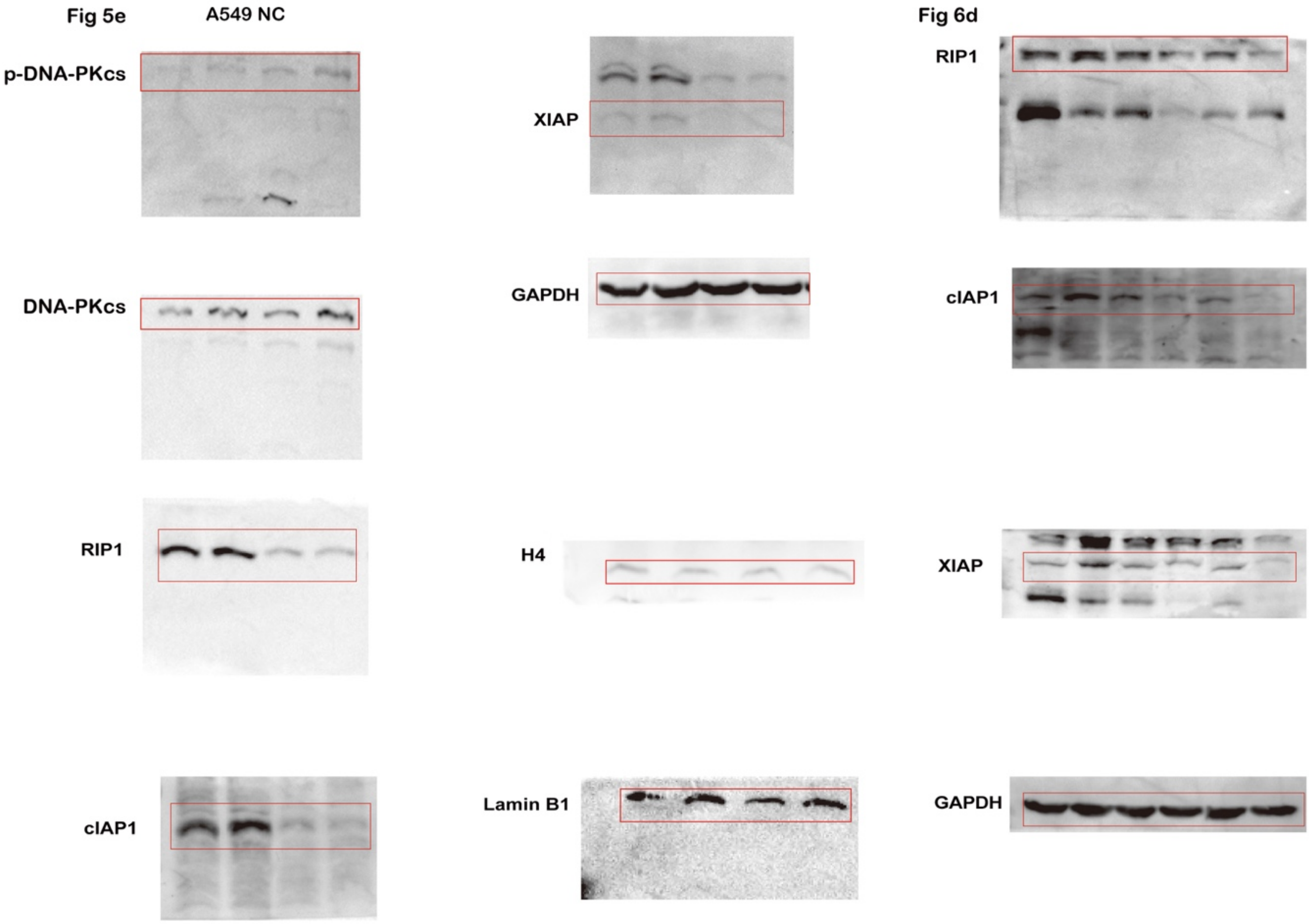

Fig S6 continued

Fig S3b

A549 H4 KD 2#

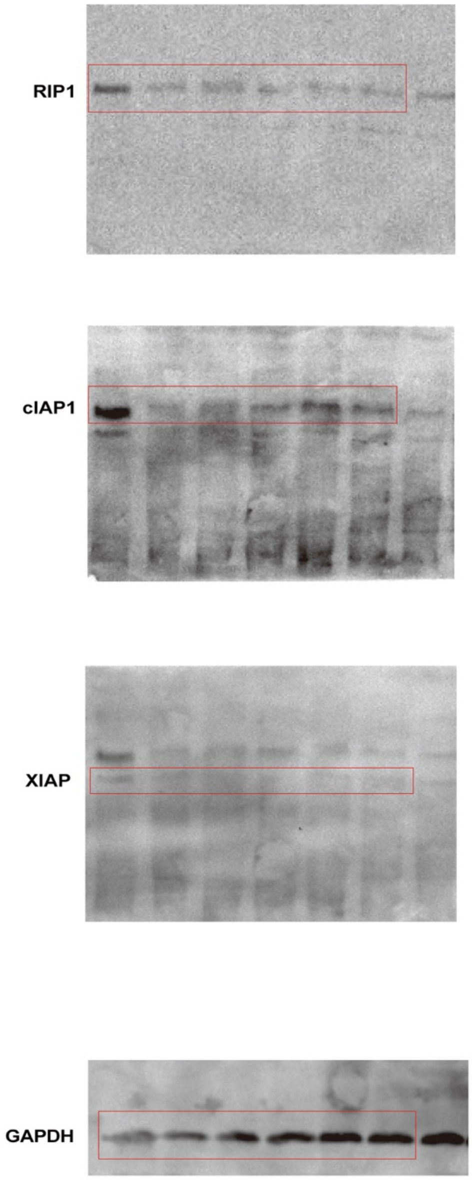

Fig S3c

A549 H4 KD 2#

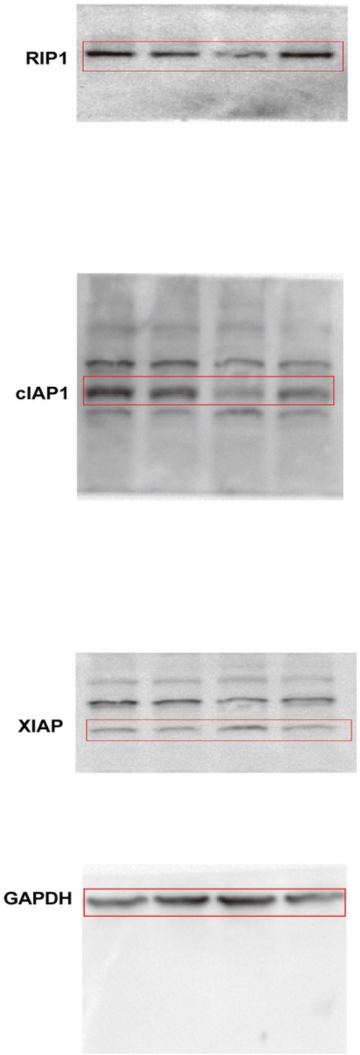

Fig S3d

A549 H4 KD 2#

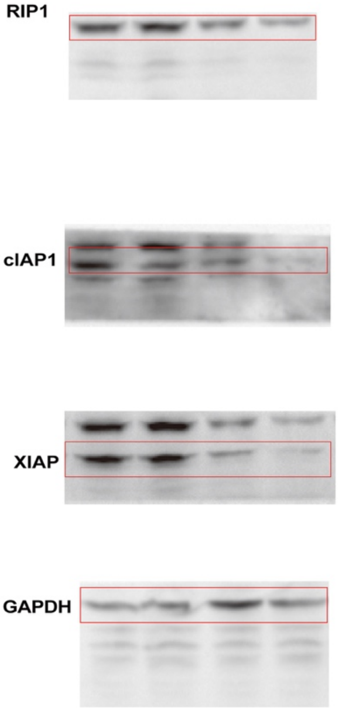

Fig S5

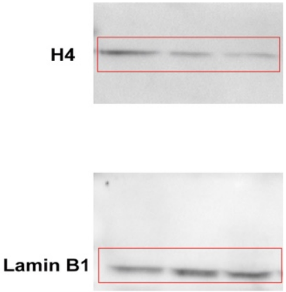

Supplement: Supplementary Methods,Table and Figures [file srep41715-s1.pdf]
